# The Crystal Structure and Luminescence Behavior of Self-Activated Halotungstates Ba_3_WO_5_Cl_2_ for W-LEDs Applications

**DOI:** 10.3390/nano15040311

**Published:** 2025-02-18

**Authors:** Liuyang Zhang, Shijin Zhou, Jiani Meng, Yuxin Zhang, Jiarui Zhang, Qinlan Ma, Lin Qin, Man Luo

**Affiliations:** School of Microelectronics and School of Integrated Circuits (Jiangsu Key Laboratory of Semi. Dev. & IC Design, Package and Test), Nantong University, Nantong 226019, China

**Keywords:** self-activated halotungstate, luminescence properties, thermal quenching

## Abstract

The self-activated halotungstate Ba_3_WO_5_Cl_2_ was successfully synthesized using a high-temperature solid-state method. X-ray diffraction analysis (XRD) confirmed the formation of a single-phase compound with a monoclinic crystal structure, ensuring the material’s purity and structural integrity. The luminescence properties of Ba_3_WO_5_Cl_2_ were thoroughly investigated using both optical and laser-excitation spectroscopy. The photoluminescent excitation (PLE) and emission (PL) spectra, together with the corresponding decay curves, were recorded across a broad temperature range, from 10 K to 480 K. The charge transfer band (CTB) of the [WO_5_Cl] octahedron was clearly identified in both the PL and the PLE spectra under ultraviolet light excitation, indicating efficient energy transfer within the material’s structure. A strong blue emission could be detected around 450 nm, which is characteristic of the material’s luminescent properties. However, this emission exhibited thermal quenching as the temperature increased, a common phenomenon where the luminescence intensity diminishes due to thermal effects. To better understand the thermal quenching behavior, variations in luminescence intensity and decay time were analyzed using a straightforward thermal quenching model. This comprehensive study of Ba_3_WO_5_Cl_2_ luminescent properties not only deepens the understanding of its photophysical behavior but also contributes to the development of novel materials with tailored optical properties for specific technological applications.

## 1. Introduction

Luminescent materials play a crucial role in diverse modern technologies, such as display systems, lighting, biomedical imaging, and security applications. These materials, which emit light upon stimulation by external energy sources, are divided into two main categories: phosphorescent and fluorescent materials. Phosphorescent materials continue to emit light for some time after the energy source is discontinued, which makes them ideal for ‘glow-in-the-dark’ applications. Conversely, fluorescent materials instantly emit light upon light exposure, which makes them suitable for high-brightness and energy-efficient applications like LED lighting and LCD displays [1].

Fluorescent materials are typically activated by rare earth (RE) ions, such as Eu^3+^, Tb^3+^, Tm^3+^, etc., which are known for their high emission quantum efficiency and long excited state lifetimes, both of which are essential for fluorescence decay lifetime measurements [2,3,4]. Compared to RE-activated materials, RE-free phosphors offer several advantages, including being free of RE elements, a lower cost, broader emission wavelengths, and preparation at lower temperatures [5,6]. The development of RE-free phosphors typically involves three strategies: (i) using transition metals as luminescence centers [7]; (ii) utilizing defects such as oxygen vacancies [8]; (iii) employing oxyacid compounds like vanadates and tungstates [9].

Particularly, closed-shell transition metals are known to produce broad-band emission within the visible region. These complexes typically consist of a central, highly charged transition metal ion, such as Ti^4−^, V^5−^, W^6−^ or Mo^6−^, surrounded by oxygen ions in tetrahedral or octahedral configurations [10,11,12]. Among these materials, tungstates have garnered significant attention as candidates for non-RE full-color phosphors, owing to their high light yield, substantial X-ray absorption coefficient, and excellent chemical stability [13]. Tungstate materials constitute a subcategory of the self-activated luminescent family, characterized by the emission of light in the absence of external activators. This phenomenon is attributed to the unique crystal structure and specific electronic transitions observed in these materials, which originate from anion ligands and reach the central metal ion, which exhibits a d0 electronic configuration. The intrinsic luminescence of tungstates was initially reported by G. Blasse et al. in the CaWO4 compound with a scheelite-type structure, in which the coordination of the W cation is achieved through the formation of a regular tetrahedral arrangement comprising four oxygen atoms. Subsequently, several self-activated tungstates were reported, for example, CsGa_0.333_W_1.667_O_6_ [14], A_2_W_3_O_10_ (A = Rb and Cs) [15], NaLaMgWO_6_ [16], etc. For tungstates, the ground state is represented by the ^1^A_1_ level, while the lowest energy excited state results from the splitting of the ^3^T_1_ level.

In this study, the halotungstate compound Ba_3_WO_5_Cl_2_ is presented as a novel self-activated phosphor. The luminescent properties of the synthesized material were meticulously analyzed in conjunction with its crystal structure, offering a comprehensive understanding of its photophysical behavior. Detailed characterization of the temperature-dependent luminescence provided key insights into the material’s performance across various thermal conditions. The results demonstrated that Ba_3_WO_5_Cl_2_ exhibits promising luminescent properties and can be considered a viable candidate for LED phosphors.

## 2. Materials and Methods

### 2.1. Synthesis of Ba_3_WO_5_Cl_2_

The self-activated halotungstate phosphor Ba_3_WO_5_Cl_2_ was successfully prepared using a two-step solid-state method. The synthesis began with BaCO_3_, WO_3_ and BaCl_2_ as the raw materials. Initially, stoichiometric amounts of BaCO_3_ and WO_3_ were thoroughly mixed in an agate mortar to form a uniform mixture. This mixture was then subjected to calcination at 400 °C for 10 h in a crucible under atmospheric conditions. The calcination process facilitated the initial formation of intermediate compounds. Following calcination, the mixture was carefully reground to enhance homogeneity and then sintered at 850 °C for 8.5 h in a muffle furnace, leading to the formation of Ba_2_WO_5_. Ba2WO_5_ was then weighed and thoroughly ground with a stoichiometric amount of BaCl_2_ to ensure a precise chemical composition. This final mixture underwent annealing at 750 °C for 2 h in a muffle furnace. After cooling to room temperature, the process resulted in the successful formation of the self-activated Ba_3_WO_5_Cl_2_ halotungstate.

### 2.2. Characterization

The XRD patterns were obtained using a Rigaku D/Max diffractometer (Rigaku, Tokyo, Japan) set to operate at 40 kV and 30 mA, employing Bragg–Brentano geometry with Cu-Kα1 radiation (λ = 1.5405 Å). The XRD measurements were conducted over a 2θ range of 10° to 70°, with a step increment of 0.02 ° and a scanning rate of 4.0 ° per minute, ensuring a precise resolution of the diffraction peaks and an accurate identification of the crystal structure. For the PLE and PL spectra, the phosphors were analyzed using a Perkin-Elmer LS-50B luminescence spectrometer (Perkin Elmer, Waltham, MA, USA). The luminescence properties, including the emission and decay characteristics, were thoroughly investigated across a wide temperature range of 10 to 480 K. To achieve accurate temperature control, the samples were mounted in a vacuum specimen chamber, with a continuous flow of liquid helium to maintain the desired temperature conditions. Excitation was provided by a 266 nm light source, generated from the fourth harmonic of a pulsed Nd laser, ensuring a high-energy input for efficient excitation. Dynamic luminescence data, including decay curves, were captured using a Tektronics TDS754A digital storage oscilloscope (500 MHz) (Tektronics, Beaverton, OR, USA), which allowed for precise measurement and analysis of the temporal evolution of the luminescent signals.

## 3. Results and Discussion

**Figure 1:**
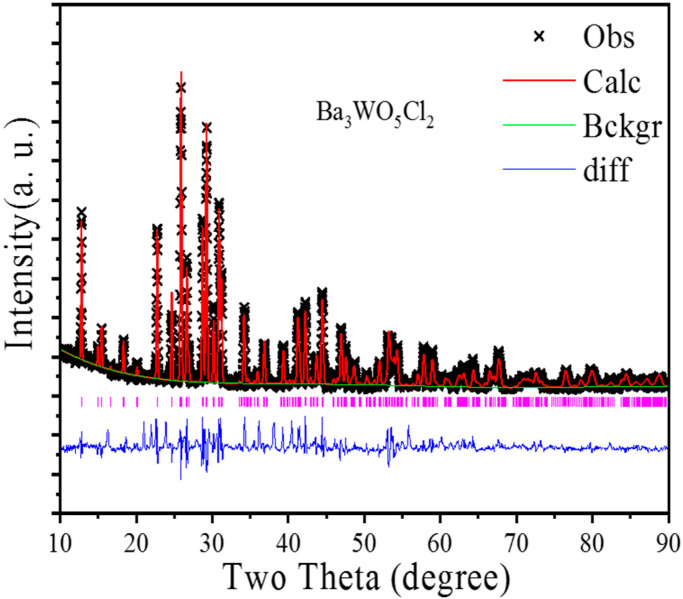
Experimental (black points) and calculated XRD patterns (red solid line) and their differences (blue solid line) for the Rietveld fit of the Ba_3_WO_5_Cl_2_ phosphor. The short vertical lines (pink lines) show the position of Bragg reflections of the calculated patterns.

**Figure 2:**
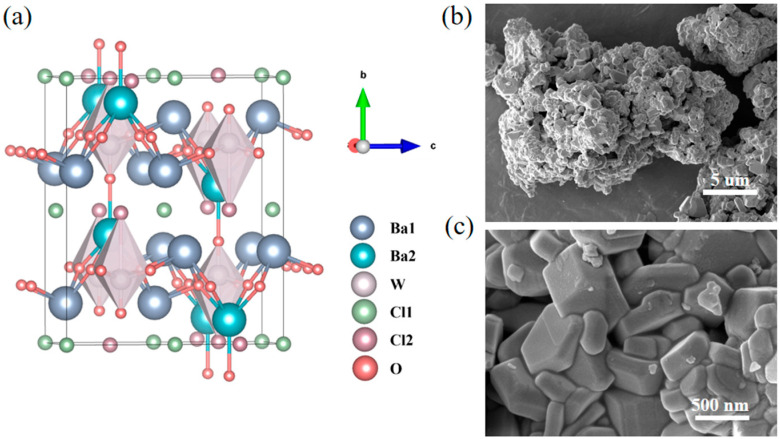
(**a**) The configuration of the atoms in crystal structure of Ba_3_WO_5_Cl_2_; (**b**,**c**) SEM images of Ba_3_WO_5_Cl_2_.

**Figure 3:**
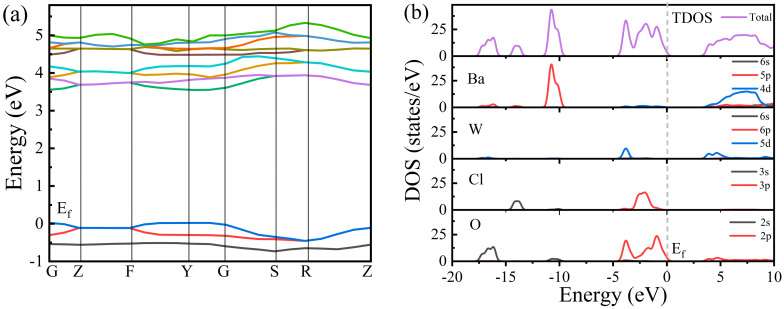
(**a**) The calculated energy band structure of Ba_3_WO_5_Cl_2_; (**b**) DOSs of Ba_3_WO_5_Cl_2_ (Fermi level at 0 energy).

Figure 1 displays the Rietveld refinement analysis of the XRD patterns for the as-synthesized Ba_3_WO_5_Cl_2_. The comprehensive crystallographic data and structural parameters obtained through the Rietveld refinement are provided in Table 1. Additionally, Table 2 offers detailed information on the positions, thermal parameters (Biso) and occupancy levels of the constituent atoms. The experimental data, indicated by black squares, aligned closely with the calculated data, represented by the red line, as validated by the goodness-of-fit indicators. Ba_3_WO_5_Cl_2_ crystallized in an orthorhombic structure with the unit cell parameters a = 5.796(2) Å, b = 13.825(2) Å, c = 11.469(2) Å, V = 919.01(38) Å^3^, and Z = 4, within the Cmcm space group (No. 63) [17], as shown in Figure 2a. Scanning electron microscopy (SEM) images of the as-prepared Ba_3_WO_5_Cl_2_ powdery sample are displayed in Figure 2b,c. The phosphors exhibited a granular morphology with a uniform particle size of 500 nm. The electronic structure of Ba_3_WO_5_Cl_2_ was further explored through first-principles calculations with the VASP code. As illustrated in Figure 3a, the calculated band structure indicates that Ba_3_WO_5_Cl_2_ exhibited a direct band gap of 3.54 eV. This band gap is within the ideal range for host materials in luminescent applications, which typically require broad band gaps between 3.0 and 6.0 eV to effectively accommodate both the ground and the excited states of luminescent ions [18]. To gain deeper insights into the electronic properties, the total and partial density of states (DOSs) of Ba_3_WO_5_Cl_2_ were calculated, as shown in Figure 3b. The analysis indicated that the top of the valence band is primarily composed of the O-2p and Cl-3p states, with minor contributions from the W-5d and Ba-5p states, extending from −5 eV up to the Fermi level (at 0 eV). The energy band from −8 eV to −20 eV is mainly formed by the Ba-5p, Cl-3s and O-2s states. Meanwhile, the conduction band, ranging from 3.54 eV to 7.60 eV, predominantly comprises the W-5d states, with slight contributions from the Ba-5p states. These results suggest that the primary host absorptions in Ba_3_WO_5_Cl_2_ can be attributed to charge transfer transitions (CT) from the O-2p and Cl-3p states to the W-5d and Ba-5p states.

Based on the band arrangement theory, the conduction band potential (*E*_CB_) and the valence band potential (*E*_VB_) of Ba_3_WO_5_Cl_2_ can be calculated using specific equations:

(1) $$E_{\mathrm{CB}}=X-E^{e}-0.5E_{g}$$

(2) $$E_{\mathrm{VB}}=X-E^{e}+0.5E_{g}$$

In these equations, *E*^e^ represents a constant value of 4.5 eV, and *E*_g_ refers to the bandgap energy, which is 3.54 eV for Ba_3_WO_5_Cl_2_. The electronegativity (X) of Ba_3_WO_5_Cl_2_ was determined by the weighted average of the electronegativities of its constituent elements:

(3) $$X\left({\mathrm{Ba}}_{3}{\mathrm{WO}}_{5}{\mathrm{Cl}}_{2}\right)=\sqrt[{6}]{{X(\mathrm{Ba})}^{3}X(W){X(O)}^{5}{X(\mathrm{Cl})}^{2}}=5.35\mathrm{eV}$$

where X(Ba) = 2.4 eV, X(W) = 4.4 eV, X(O) = 7.54 eV, and X(Cl) = 8.3 eV [19]. By applying these values, the calculated *E*_CB_ for Ba_3_WO_5_Cl_2_ is −0.92 eV, and the *E*_VB_ is 2.62 eV. These potentials provide significant information on the electronic structure and reactivity of the material.

**Figure 4:**
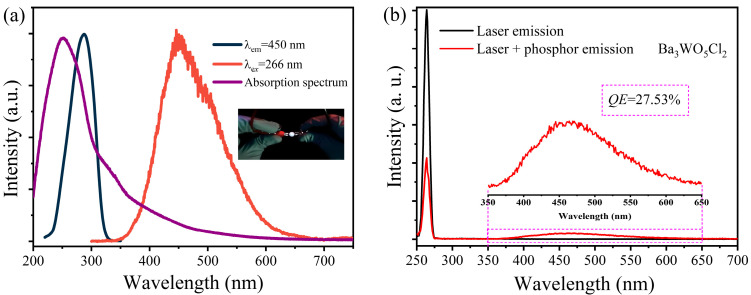
(**a**) The UV-Vis absorption spectrum and PL and PLE spectra of Ba_3_WO_5_Cl_2_; the insert shows the diagram of the device; (**b**) internal quantum efficiency of Ba_3_WO_5_Cl_2_; inset: zoomed-in fluorescence spectrum around the 460 nm peak.

Tungstates are well known for their intrinsic self-activated luminescent properties, which make them ideal candidates for various photonic applications. The typical UV-Vis absorption spectrum and PL and PLE spectra of Ba_3_WO_5_Cl_2_ were thoroughly characterized and are exhibited in Figure 4a. The UV–vis absorption spectrum showed that Ba₃WO₅Cl₂ could absorb UV light below 400 nm. The excitation of Ba_3_WO_5_Cl_2_ occurs at wavelengths ranging from 200 to 380 nm, with a prominent peak at 310 nm. Upon excitation, the material emits light at wavelengths ranging from 350 to 650 nm, with a peak emission at 450 nm. This broad emission spectrum results in the generation of bright white light, as demonstrated by the inset of Figure 4a. White LEDs were constructed by combining this white-emitting phosphor with an InGaN-based near-UV LED chip that emits at 365 nm. The intrinsic luminescence of tungstates can be primarily attributed to the presence of tetrahedral WO_4_^2−^ or octahedral WO_6_^6−^, which are responsible for the charge transfer band (CTB) from the 2p orbital of the O^2−^ ligand to the 5d orbital of the d^0^ tungsten metal ion. Specifically, the intrinsic emission of Ba_3_WO_5_Cl_2_ is attributed to the radiative decay of excitons that are self-trapped in the octahedral [WO_5_Cl]^5−^. This is due to the analogous octahedral symmetry, where a tungsten ion is surrounded by five oxygen ions and one chloride ion, creating a stable luminescent center. Otherwise, the band shape of the PL and PLE spectra of Ba_3_WO_5_Cl_2_ is in agreement with those of other known tungstate compounds, such as Y_2_WO_6_ or AgGdW_2_O_8_, which also contain octahedral [WO_6_]^6−^ [20,21]. This similarity suggests that the charge transfer mechanism in Ba_3_WO_5_Cl_2_, involving the [WO_5_Cl]^5−^ octahedron, is related to the mechanisms observed in these other tungstate compounds, providing a deeper understanding of its luminescence behavior. The CIE chromaticity diagram of Ba_3_WO_5_Cl_2_ was based on the 1931 CIE standard. The chromaticity coordinates for Ba_3_WO_5_Cl_2_ were determined to be *x* = 0.18, *y* = 0.22, placing it within the blue region of the diagram. These coordinates show that, while the phosphor primarily emits blue light, its broad emission spectrum contributes to the overall white light effect when paired with a suitable UV LED source. This makes Ba_3_WO_5_Cl_2_ a promising material for the development of white LED technology, particularly in applications.

To assess the applicability of the Ba_3_WO_5_Cl_2_ phosphor in optoelectronic devices, a UV-LED chip emitting at 266 nm was employed, as exhibited in the insert of Figure 4a. As for the results presented in Figure 4b, the quantum efficiency (QE) of the phosphor was calculated using the equation below [22]:

(4) $$\mathrm{QE}=\frac{\int L_{s}}{\int E_{R}-\int E_{s}}$$

where *L_S_* denotes the integrated emission spectrum of Ba_3_WO_5_Cl, whereas *E_S_* and *E_R_* represent the integrated excitation spectra in the presence and absence of the Ba_3_WO_5_Cl coating, respectively. Using this formula, the QE of Ba_3_WO_5_Cl_2_ was determined to be approximately 27.53%. This QE value is within the mid-range of those reported for other white light-emitting materials, indicating that the synthesized tungstate possesses promising potential for practical applications in fields such as lighting, display technologies and optoelectronic devices [23,24].

**Figure 5:**
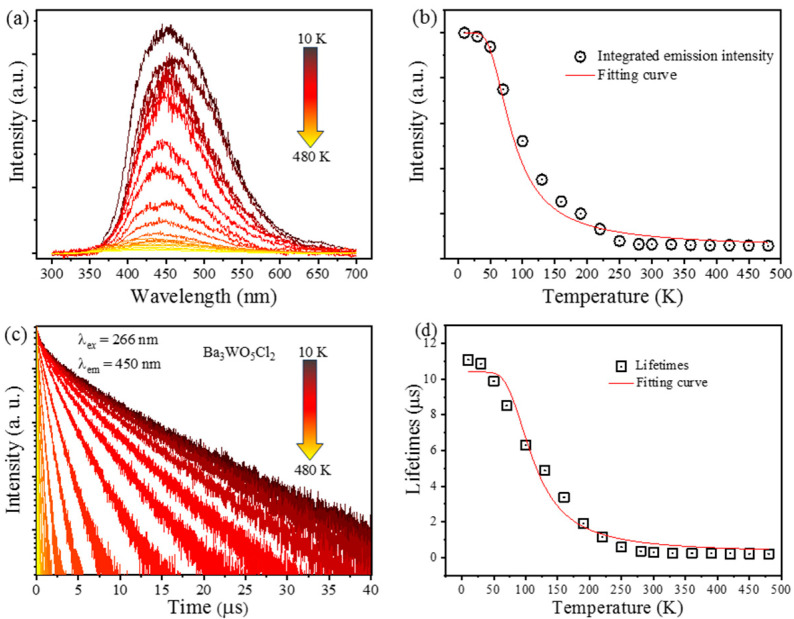
(**a**) Normalized temperature-dependent emission spectra of Ba_3_WO_5_Cl_2_; (**b**) calculated emission intensities of Ba_3_WO_5_Cl_2_; (**c**) normalized temperature-dependent decay curves of Ba_3_WO_5_Cl_2_; (**d**) relationship between average lifetime and temperature for Ba_3_WO_5_Cl_2_.

The temperature-dependent emission spectra of phosphors are crucial for evaluating their thermal stability and overall efficiency, particularly in applications like LED lighting and display technologies. Understanding how these spectra behave at varying temperatures is essential for optimizing phosphors to ensure consistent color output and brightness, even under fluctuating thermal conditions. This knowledge directly contributes to the development of reliable and durable lighting solutions. Figure 5a presents the PL spectra of the Ba_3_WO_5_Cl_2_ phosphor at various temperatures.

As shown, the emission spectra exhibited a noticeable change as the temperature increased, which was further quantified in Figure 5b, where the emission intensities are plotted against temperature. A noticeable decrease in emission intensity occurred with the increasing temperature, a phenomenon attributed to thermal quenching. Thermal quenching occurs when the thermal energy provided to the system activates non-radiative processes, leading to the intersection of the ground and excited states and a consequent reduction in luminescence. This process is a well-documented phenomenon in the literature [25,26] and is particularly important for materials used in high-temperature environments. The emission spectra of Ba_3_WO_5_Cl_2_ were modeled as a superposition of broad-band emission intensities, which allowed for a detailed analysis of how thermal effects influence the overall luminescence.

The photoluminescence intensity as a function of temperature is commonly described using a modified Arrhenius equation, which accounts for the non-radiative processes that become more prominent at elevated temperatures [27]:

(5) $$I(T)=\frac{I_{0}}{1+\gamma \exp(\frac{-{\text{∆}E}_{a}}{\mathrm{kT}})}$$

in the context of the modified Arrhenius equation, ∆*E*_a_ represents the activation energy for thermal quenching, *I*_0_ denotes the initial emission intensity at the reference temperature, *γ* is a constant associated with the ratio of radiative to non-radiative carrier lifetimes, and *k* is the Boltzmann constant, whose value is 8.629 × 10^−5^ eV. For Ba_3_WO_5_Cl_2_, ∆*E*_a_ was determined to be 24.2 meV, while γ was found to be 22.8. Meanwhile, the quenching temperature, which is the point where the emission intensity drops to half of its initial value at 10 K (I_10 K_), is 160 K for Ba_3_WO_5_Cl_2_. The sharper decline in intensity as the temperature rose can be attributed to either a lower ∆*E*_a_ or a higher γ, indicating that the material is more susceptible to thermal quenching. The relatively high γ value observed for Ba_3_WO_5_Cl_2_ suggests a shorter non-radiative lifetime of the carriers, which contributed to the rapid decline in emission intensity as the temperature rose. This behavior was further compounded by the significant Stokes’ shift observed for Ba_3_WO_5_Cl_2_, which typically leads to enhanced thermal quenching. Stokes’ shift refers to the difference between the absorption and the emission wavelengths, and a larger shift generally indicates that a material is more prone to non-radiative relaxation processes, which reduces its overall luminescence efficiency.

Additionally, as the temperature increased, the emission bands shifted noticeably from 530 nm to 510 nm, indicating a blue shift in the spectra. Generally, as the temperature of crystalline substances rises, their lattices expand, which generally leads to a reduction in the crystal-field splitting parameter [Δ(Dq)] [28]. According to theoretical principles, a significant decrease in this parameter would favor radiative transitions at longer wavelengths, resulting in a redshift of the emitted light. However, the blue shift observed for Ba_3_WO_5_Cl_2_ suggests a different underlying mechanism. This blue shift may be attributed to thermal crossing between two excited states within the tungstate groups [29]. Such thermal interactions can alter the energy levels involved in radiative transitions, leading to emission at shorter wavelengths as the temperature increases. This phenomenon is somewhat unusual and contrasts with the more commonly observed redshift.

In addition to the spectral shift, the thermal quenching behavior of Ba_3_WO_5_Cl_2_ was further analyzed through temperature-dependent decay curves, as illustrated in Figure 5c. The decay curve at 10 K shows a slightly non-exponential behavior, which is likely due to the presence of multiple luminescent centers within the material. Such anomalous decay behavior has been previously reported for other self-activated phosphors, such as NaWO_2_PO_4_, as reported by Blasse et al. [30]. This suggests that Ba_3_WO_5_Cl_2_ may possess a complex luminescent environment, where different emission centers contribute to the overall decay profile. To quantitatively assess the decay behavior, the mean lifetime of the emission can be calculated using the following equation [31]:

(6) $$\tau_{\mathrm{avg}}=\frac{\int tI\left(t\right)dt}{\int I\left(t\right)dt}$$

The measured luminescence lifetimes of Ba_3_WO_5_Cl_2_ exhibited a significant reduction at elevated temperatures, with the average lifetimes decreasing from 11.7 μs at 10 K to 0.52 μs at 420 K. This drastic shortening is primarily due to the increased probability of non-radiative transitions as the temperature rises, which compete with radiative processes and diminish the overall emission efficiency.

The relationship between average lifetime and temperature is shown in Figure 5d, highlighting the strong correlation between increased temperature and decreased luminescence lifetime. This behavior is indicative of thermal quenching, where higher thermal energy facilitates non-radiative decay pathways, thereby reducing the effective emission duration. To further quantify the thermal quenching effect, Δ*E* for Ba_3_WO_5_Cl_2_ was calculated by analyzing the temperature dependence of the emission lifetime. The following equation was used [32]:

(7) $$\tau (T)=\frac{\tau r}{1+[\tau r/\tau nr]\exp(-\Delta E/kT)}$$

where *τ*(*T*) refers to the temperature-dependent lifetime, *τr* and *τnr* are the radiative and non-radiative lifetimes, respectively, and k refers to the Boltzmann constant. By fitting the experimental data to this model, ∆*E*_a_ for thermal quenching in Ba_3_WO_5_Cl_2_ was determined. The fitting results obtained from the temperature-dependent lifetimes of Ba_3_WO_5_Cl_2_ are consistent with the values derived from the temperature-dependent emission intensities, reinforcing the reliability of the model. This consistency not only validates the calculated ΔE but also underscores the importance of understanding the interplay between radiative and non-radiative processes when determining the thermal stability of luminescent materials. Meanwhile, these findings offer valuable insights into the thermal behavior of Ba_3_WO_5_Cl_2_, indicating that while the material exhibits promising luminescent properties at lower temperatures, its performance at higher temperatures is significantly impacted by thermal quenching. This understanding is essential for the practical application of Ba_3_WO_5_Cl_2_ in devices that may operate under varying thermal conditions, such as high-power LEDs, lasers, and other optoelectronic systems. The absolute sensitivity, S_a_, and the relative sensitivity, S_r_, were calculated. It was established that the relative sensitivity, S_r_, reached a maximum value of 1.098% K^−1^ at 150 K. In contrast, the absolute sensitivity, S_a_, reached a maximum value of 0.021 K^−1^ at 300 K, an outcome which is expected to prove useful for future applications. Future work may focus on optimizing the composition or structure of Ba_3_WO_5_Cl_2_ to enhance its thermal stability and extend its applicability across a broader temperature range.

## 4. Conclusions

The self-activated halotungstate Ba_3_WO_5_Cl_2_ was successfully synthesized using a solid-state reaction method. XRD analysis confirmed the formation of a single-phase material, which is well-aligned with the orthorhombic structure and belongs to the Cmcm (63) space group. The electronic structure of Ba_3_WO_5_Cl_2_ was further investigated through first-principles calculations, revealing a direct band gap of 3.54 eV. This band gap is primarily attributed to charge transfer transitions from the O-2p and Cl-3p states to the W-5d and Ba-5p states, underscoring the material’s potential as an efficient phosphor. Photoluminescence studies demonstrated that Ba_3_WO_5_Cl_2_ shows excellent luminescence properties, achieving a maximum QE value of 27.53% under the excitation with near-UV light. This high QE, combined with the material’s strong luminescence, makes it particularly suitable for use in W-LEDs when paired with near-UV chips. Additionally, thermoluminescence studies revealed a blue shift in the emission peaks as the temperature increased, which was likely due to thermal crossing between two excited states within the tungstate groups. This unique thermal behavior further highlights the material’s versatility and potential for application in temperature-sensitive environments. Overall, the results indicate that Ba_3_WO_5_Cl_2_ is a promising candidate for the development of W-LEDs utilizing near-UV chips. Its combination of strong luminescence, suitable electronic structure, and favorable thermal properties proposes this novel self-activated tungstate as an excellent material for advanced optoelectronic applications. Future research could focus on optimizing its performance and exploring additional applications in other photonic devices.

## Figures and Tables

**Table 1:** The crystal parameters of Ba_3_WO_5_Cl_2_.

Formula	Ba_3_WO_5_Cl_2_	Refined Ba_3_WO_5_Cl_2_
Symmetry	Orthorhombic	Orthorhombic
Space group#	C m c m (63)	C m c m (63)
a/Å	5.796 (2)	5.783 (9)
b/Å	13.825 (2)	13.836 (7)
c/Å	11.469 (2)	11.466 (2)
Z	4	4
V/Å^3^	919.01 (38)	919.14 (22)
R_p_	-	0.0234
R_wp_	-	0.0164
χ^2^	-	1.250

**Table 2:** The detailed refined parameters of the atomic coordinates and occupancy of Ba_3_WO_5_Cl_2_.

Atom	Ox.	Wyck.	Site	x/a	y/b	z/c	U [Å^2^]
Ba1	2	8f	m	1/2	0.1521 (1)	0.4555 (1)	0.0025 (6)
Ba2	2	4c	m2m	1/2	0.4082 (1)	1/4	0.0025 (6)
W1	6	4c	m2m	0	0.2457 (1)	1/4	0.0025 (6)
Cl1	−1	4a	2/m	0	0	0	0.0025 (6)
Cl2	−1	4c	m2m	0	0.4947 (5)	1/4	0.0025 (6)
O1	−2	16h	1	0.2242(13)	0.2809 (5)	0.1363 (6)	0.0025 (6)
O2	−2	4c	m2m	0	0.119 (2)	1/4	0.0025 (6)

## Data Availability

All data from this study are available from the corresponding author upon request.
